# The relationship between pain management and psychospiritual distress in patients with advanced cancer following admission to a palliative care unit

**DOI:** 10.1186/s12904-015-0067-2

**Published:** 2015-12-02

**Authors:** Ya-Ping Lee, Chih-Hsun Wu, Tai-Yuan Chiu, Ching-Yu Chen, Tatsuya Morita, Shou-Hung Hung, Sin-Bao Huang, Chia-Sheng Kuo, Jaw-Shiun Tsai

**Affiliations:** Division of Family Medicine, Taipei Hospital, Ministry of Health and Welfare, New Taipei City, Taiwan; Department of Family Medicine, Hospice and Palliative Care Unit, College of Medicine and Hospital, National Taiwan University, 7 Chung-Shan South Road, Taipei, Taiwan; Department of Psychology, National Chengchi University, Taipei, Taiwan; Division of Geriatric Research, Institute of Population Health Science, National Health Research Institutes, Ju-Nan, Taiwan; Palliative and Supportive Care Division, Seirei Mikatahara Hospital, Mikatahara, Kita, Hamamatsu, Japan; Department of Community and Family Medicine, National Taiwan University Hospital Yun-Lin Branch, Yun-Lin, Taiwan; Department of Palliative Care, Changhua Christian Hospital, Changhua, Taiwan; Department of Family Medicine, Changhua Christian Hospital, Changhua, Taiwan; Center for Complementary and Integrated Medicine, National Taiwan University Hospital, Taipei, Taiwan

**Keywords:** Cancer pain, Psychospiritual distress, Advanced cancer, Hospice palliative care

## Abstract

**Background:**

Although many cross-sectional studies have demonstrated the association between cancer pain and psychospiritual distress, the time-dependent relationship has not been fully explored. For that reason, this study aims to investigate the time-dependent relationship between psychospiritual distress and cancer pain management in advanced cancer patients.

**Methods:**

This is a prospective observational study. Two hundred thirty-seven advanced cancer patients were recruited from a palliative care unit in Taiwan. Demographic and clinical data were retrieved at admission. Pain and psychospiritual distress (i.e.: anxiety, depression, anger, level of family and social support, fear of death) were assessed upon admission and one week later, by using a “Symptom Reporting Form”. Patients were divided into two groups according to the pain status one week post-admission (*improved* versus *not improved* groups).

**Results:**

One hundred sixty-three (68.8 %) patients were assigned to the *improved* group, and 74 (31.2 %) patients were assigned to the *not improved* group. There were no differences in the psychospiritual variables between groups upon admission. In overall patients, all psychospiritual variables improved one week post-admission, but the improvement of depression and family/social support in the *not improved* group was not significant. Consistent with this, for depression scores, there was a statistically significant *pain group x time* interaction effect detected, meaning that the pain group effect on depression scores was dependent on time.

**Conclusions:**

We demonstrated a time-dependent relationship between depression and pain management in advanced cancer patients. Our results suggest that poor pain management may be associated with intractable depression. The inclusion of interventions that effectively improve psychospiritual distress may contribute to pain management strategies for advanced cancer patients.

## Background

Moderate to severe pain affects 70–80 % of advanced cancer patients [1]. Although the World Health Organization’s (WHO’s) analgesic ladder has been reported to provide adequate pain relief in 80–90 % of cancer patients [2, 3], more recent reports have brought this percentage into question. In fact, pain may be undertreated in as many as 43 % of cancer patients [4]. Regardless of pharmacological treatment, most advanced cancer patients still experience pain and report that their quality of life is significantly compromised [5, 6]. Thus, pain management is still unsatisfactory in this patient population, and remains a core issue in cancer patient care.

Advanced cancer patients often exhibit symptoms of weakness, pain, anorexia, and cachexia [7]. There is a high prevalence of these symptoms in advanced cancer patients regardless of the primary cancer site [7]. We have reported that 81 % of patients with advanced cancer admitted to a palliative care unit in Taiwan reported having pain [7]. Furthermore, we have reported that the constellation of symptoms associated with advanced cancer can be grouped according their pattern of expression (i.e.: symptom patterns) at the end stage of life [8]. Pain and depression severity followed the same “decrease-static” symptom pattern, which is characterized by only an initial improvement in symptoms after admittance into palliative care, followed by a maintenance of that level until death [8]. Therefore, it is crucial to make great progress in pain management in these patients.

Although advanced cancer patients experience both psychological and physical pain, treatment is mostly targeted at alleviating the physical symptoms [9]. However, cultural and psychosocial factors can hinder pain management [10]. Psychological factors such as depression, anxiety and a fear of catastrophes are associated with more severe pain [11]. Indeed, cancer pain can become intractable, particularly in the presence of psychological distress [12]. Unrecognized psychosocial variables that cause distress can worsen pain severity and increase the use of medications [12]. Besides, spiritual distress can also aggravate the physical/psychological symptoms of cancer patients [13]. Thus, cancer pain is a multidimensional phenomenon and a complex subjective experience.

Advanced cancer patients often experience suffering of the whole person. Thus, in order to better manage cancer pain, it is important to consider not only biomedical factors but also the level of psychosocial and spiritual distress of the patient [14]. There have several cross-sectional studies reporting that pain is associated with psychosocial distress [15–17]. Despite literatures reported that psychological distress complicates cancer pain considerably [12], there is lack of quantitative evidence showing the time-dependent relationship between pain and psychospiritual distress in advanced cancer patients. The aim of this study was to examine the time-dependent relationship between pain management and psychospiritual distress in advanced cancer patients admitted to a palliative care unit.

## Methods

### Study design, patients and palliative care setting

This is a prospective observational study. Participants were selected from patients with advanced cancer, not responsive to any caner therapy administrated by oncologists and consecutively admitted to the Palliative Care Unit of the National Taiwan University Hospital between October 2006 and December 2007. All patients provided informed consent. The conscious levels of patients were divided into six categories by primary care physicians: alertness (normal response to orders), lethargy (sleepy but normal response to orders), obtundation (slow response to orders), delirium (confusion), stupor (near-unconsciousness) and coma (unconsciousness). The inclusion criteria is that the individual’s level of consciousness had to be clear enough (alert or lethargic consciousness) to report symptoms both on admission and one week after admission. The participants were under active, total care, provided by a multidisciplinary team of physicians, nurses, psychologists, social workers, clinical Buddhist chaplains, and volunteers. The physicians managed the patients’ symptoms by pharmacologic and non-pharmacologic strategies and coordinated the care team. The nurses provided routine nursing care. The psychologists provided psychotherapy, such as cognitive behavioral therapy, positive thinking, problem solving, relaxation strategies and so on. The social workers provided psychosocial and financial support. The clinical Buddhist chaplains provided spiritual support including life review, fulfillment of spiritual needs, and prayer for the patients and their family. Team meetings were held on a weekly basis. This study was approved by the ethical committee at the National Taiwan University Hospital.

### Symptom assessment and data collection

Patient demographics (age, gender, primary site of cancer, and survival days) were obtained from routine records. The symptom assessment tool was a “Symptom Reporting Form” which was used to assess physical, psychosocial and spiritual distress using different scale systems [7, 8]. Information was gathered from the “Symptom Reporting Forms” at the time of admission and one week after admission. Pain scores were rated on a 10 point likert scale of 0–10 (0 = none, and 10 = extreme). A psychosocial evaluation was conducted by the psychologists to assess the severity of depression, anxiety, and anger on a scale of 1–5 (1, almost none; 2, mild; 3, moderate; 4, severe; 5, extreme); the degrees of family and social support were rated on a scale of 1–6 (1, extreme not fit; 2, not fit; 3, somewhat not fit; 4, somewhat fit; 5, fit; 6, very fit). Clinical Buddhist chaplains conducted the fear of death assessment on a scale of 1–5 (1, very little fear, peaceful, and happy; 2, little fear but can be managed and no company required; 3, fear and company is required but the fear can be managed; 4, extreme fear, company required, and fear of sleeping at night; 5, confusion, losing autonomy, and rejecting help from others) [18].

The “Symptoms Reporting Form” was designed by experienced specialists and has been used in our previous studies [7–9, 19–21]. A content validity index was used to determine the validity of the structured questionnaire and yielded an index of 0.96. A pilot study further confirmed the instrument’s content validity and ease of application [19]. Death fear scale in the study was also designed by experienced specialists and has been used in our previous studies [18, 21, 22]. A content validity index was used to determine the validity of the structured questionnaire and yielded a score of 0.93. Ten volunteers (bereaved family members) filled out the questionnaire to confirm the questionnaire’s face validity and ease of application [22].

### Statistical analysis

Participants were assigned to one of two groups based on whether their pain scores were lower one week after admission or not (*improved* versus *not improved* groups). The patients whose pain scores reported one week after admission were lower than those on admission were assigned to the *improved* group; the other patients were assigned to the *not improved* group. Descriptive measures of data were summarized as frequencies and percentages for categorical and interval variables, and mean ± standard deviation (SD) for non-categorical variables. The *t* test and mixed designed analysis of variance (ANOVA) with one *between-subject* factor “pain group” and one *within-subject* factor “time” were used to explore the relationships between cancer pain and psychospiritual factors. Statistical significance was defined as a *p* value less than 0.05. All data were analyzed by using SAS 9.2 statistical software.

## Results

Based on the inclusion criteria, 237 patients were enrolled in this study. There were 111 (46.8 %) men and 126 (53.2 %) women. The mean age of all patients was 64.05 ± 13.87 years. The most common primary cancer sites included lung (19.4 %), liver (17.7 %), and colon/rectum (8.9 %). The mean survival was 39.54 ± 47.72 days. The median survival was 22.5 days (ranging from 7 to 418 days). One week after admission, 163 (68.8 %) patients reported an improvement in cancer pain (*improved* group) and 74 (31.2 %) patients reported no improvement in cancer pain (*not improved* group). The demographic and diagnostic data were not significantly different between the two groups (Table 1).

**Table 1:** Descriptive statistics of demographic and primary cancer sites in different pain control groups

	Group by pain control status			
Variable	Improved (n = 163)	Not improved (n = 74)	Statistics (t-test/χ2test)	*p*
Age (years)	63.62 ± 13.76	64.73 ± 13.99	−0.57	.567
Survival (days)	40.98 ± 40.97	32.58 ± 37.62	1.41	.161
Gender			0.01	.924
Male	76(46.6 %)	35(47.3 %)		
Female	87(53.4 %)	39(52.7 %)		
Primary Cancer Site			2.53	.960
Lung	31 (19.0 %)	15 (20.3 %)		
Liver	30 (18.4 %)	12 (16.2 %)		
Colon and rectum	14 (8.6 %)	7 (9.5 %)		
Head and neck	13 (8.0 %)	4 (5.4 %)		
Breast	11 (6.7 %)	3 (4.1 %)		
Stomach	10 (6.1 %)	7 (9.5 %)		
Pancreas	9 (5.5 %)	3 (4.1 %)		
Cervix/uterine	6 (3.7 %)	3 (4.1 %)		
Others	39 (23.9 %)	20 (27.0 %)		

Table 2 compares the pain scores and assessments of psychospiritual distress between the two groups at each assessment time point. At admission, pain scores were significantly higher in the group that would later report an improvement in pain one week later (5.49 ± 2.10 vs 2.20 ± 2.48, *p* < 0.001). However, measures of the psychospiritual variables were not significantly different between the two groups. One week after admission, pain scores were no longer significantly different between the two groups. It is important to note, however, that depression scores were significantly higher in the *not improved* group one week after admission (*p* = 0.016). Table 3 compares the time-dependent assessment of pain and psychospiritual distress between the two assessment time points for each group. One week after admission, a significant improvement in pain scores and all the psychospiritual distress parameters was reported by the *improved* group (all *p’s* < 0.05). However, in the *not improved* group, the improvement of depression and family/social support was not significant.

**Table 2:** Descriptive statistics of psychosocial spiritual variables in different pain control groups

	Group by pain control status					
	Improved		Not improved			
Variable	N	Mean ± SD	N	Mean ± SD	*t*_*(df)*_	*p*
At admission						
Pain	163	5.49 ± 2.10	74	2.20 ± 2.48	10.54_*(235)*_	<.001*
Anxiety	156	2.35 ± 0.98	72	2.29 ± 0.86	0.40_*(226)*_	.686
Depression	156	2.29 ± 1.02	73	2.32 ± 1.01	−0.19_*(227)*_	.853
Anger	155	1.63 ± 0.88	72	1.76 ± 0.99	−1.01_*(225)*_	.312
Family support	157	4.61 ± 1.05	73	4.48 ± 1.04	0.89_*(228)*_	.374
Social support	157	4.46 ± 1.22	73	4.29 ± 1.22	1.03_*(228)*_	.305
Fear of death	143	2.78 ± 0.75	68	2.79 ± 0.78	−0.16_*(209)*_	.873
1 week after admission						
Pain	163	2.37 ± 1.45	74	2.58 ± 2.65	−0.63_*(235)*_	.530
Anxiety	153	2.04 ± 0.92	72	2.08 ± 0.75	−0.35_*(223)*_	.723
Depression	153	1.90 ± 0.97	73	2.25 ± 1.04	−2.44_*(224)*_	.016*
Anger	151	1.42 ± 0.76	72	1.63 ± 0.88	−1.75_*(221)*_	.081
Family support	156	4.72 ± 1.01	73	4.55 ± 1.00	1.23_*(227)*_	.219
Social support	156	4.62 ± 1.22	73	4.37 ± 1.11	1.50_*(227)*_	.136
Fear of death	140	2.38 ± 0.80	70	2.59 ± 0.81	−1.76_*(208)*_	.081

*significant at 0.05 level

**Table 3:** Descriptive statistics of psychosocial spiritual variables at different time points

Variable		At admission	1 week after admission	*t*_*(df)*_	*p*
Improved					
Pain	163	5.49 ± 2.10	2.37 ± 1.45	21.98_*(162)*_	<.001*
Anxiety	153	2.33 ± 0.98	2.04 ± 0.92	4.36_*(152)*_	<.001*
Depression	153	2.27 ± 1.01	1.90 ± 0.97	5.97_*(152)*_	<.001*
Anger	151	1.62 ± 0.87	1.42 ± 0.76	3.52_*(150)*_	.001*
Family support	156	4.61 ± 1.05	4.72 ± 1.01	−2.40_*(155)*_	.018*
Social support	156	4.46 ± 1.22	4.62 ± 1.22	−3.64_*(155)*_	<.001*
Fear of death	139	2.76 ± 0.75	2.39 ± 0.79	7.44_*(138)*_	<.001*
Not improved					
Pain	74	2.20 ± 2.48	2.58 ± 2.65	−4.13_*(73)*_	<.001*
Anxiety	72	2.29 ± 0.86	2.08 ± 0.75	2.42_*(71)*_	.018*
Depression	73	2.32 ± 1.01	2.25 ± 1.04	0.82_*(72)*_	.415
Anger	72	1.76 ± 0.99	1.63 ± 0.88	2.44_*(71)*_	.017*
Family support	73	4.48 ± 1.04	4.55 ± 1.00	−1.40_*(72)*_	.167
Social support	73	4.29 ± 1.22	4.37 ± 1.11	−1.10_*(72)*_	.276
Fear of death	68	2.79 ± 0.78	2.60 ± 0.79	2.14_*(67)*_	.036*

*significant at .05 level

**Fig. 1:**
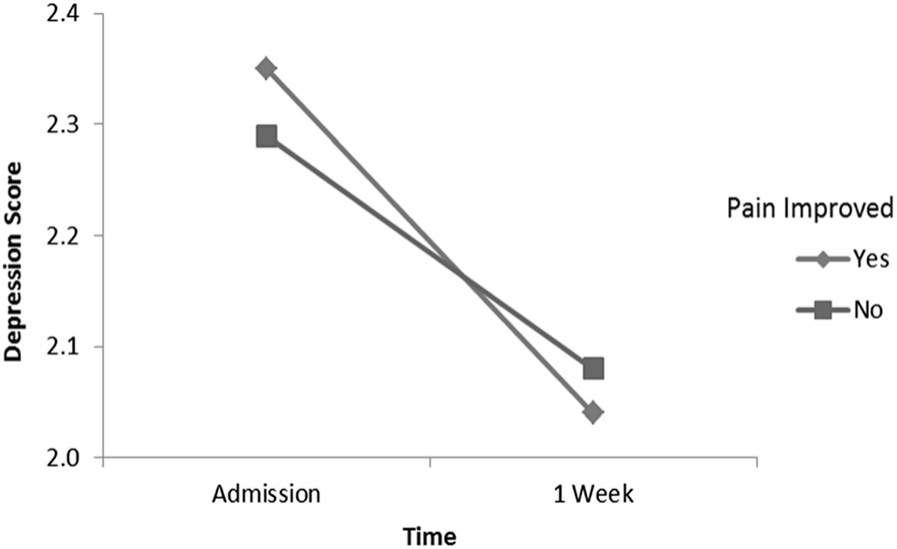
Interaction of pain control group and time on depression

We used a mixed designed ANOVA, with one between subject factor “pain group” and one within subject factor “time”, to examine the relationships between cancer pain and the variables used to reflect psychospiritual distress. No main effect of pain control status was detected for any of the factors of psychospiritual distress. However, a main effect of time was observed for all the psychospiritual factors (all *p’s* < 0.05), indicating that, being in palliative care for one week, patients reported an improvement in psychospiritual distress (Table 4). Finally, for depression scores, there was a statistically significant *pain group x time* interaction effect detected, meaning that the pain group effect on depression scores was dependent on time (*p* = 0.005) (Table 4 and Fig. 1).

**Table 4:** Interaction of pain control group and time on psychosocial spiritual distress

	Pain group		Time		Pain group x time	
Variable	*F*_*(df1,df2)*_	*p*	*F*_*(df1,df2)*_	*p*	*F*_*(df1,df2)*_	*p*
Anxiety	0.01_*(1,223)*_	.969	19.29_*(1,223)*_	<.001*	0.49_*(1,223)*_	.484
Depression	2.18_*(1,224)*_	.141	16.83_*(1,224)*_	<.001*	7.89_*(1,224)*_	.005*
Anger	2.33_*(1,221)*_	.129	14.04_*(1,221)*_	<.001*	0.36_*(1,221)*_	.548
Family support	1.18_*(1,227)*_	.278	5.57_*(1,227)*_	.019*	0.36_*(1,227)*_	.548
Social support	1.65_*(1,227)*_	.200	8.67_*(1,227)*_	.004*	0.90_*(1,227)*_	.344
Fear of death	1.38_*(1,205)*_	.241	35.16_*(1,205)*_	<.001*	3.68_*(1,205)*_	.056

*significant at .05 level

## Discussion

For overall patients, we demonstrated that psychospiritual distress improved under our active total care. In the *improved* group, all parameters of psychospiritual distress were simultaneously significantly ameliorated. However, the improvement of certain psychosocial variables was not significant in the *not improved* group. This effect was particularly dramatic for depression; while other measures of psychological distress, such as anxiety and anger, improved significantly in the *not improved* group, depression scores did not. In the *improved* group, however, depression improved significantly within one week following admission. To our knowledge, this is the first study to report that improvement in cancer pain is associated with an improvement in depression.

It is very interesting that in the *improved* group pain was significantly ameliorated one week after admission even though pain scores were significantly higher upon admission in these individuals. Since the severities of psychospiritual distress of the two groups at admission were similar, physical distress may contribute to the significant difference of pain severities in two groups at admission. Consequently, the outcome that pain in the *improved* group significantly improved may result from that most physical distress were relieved by pharmacological therapy such as opioid analgesics. This is consistent with our previous observations that better pain management could be achieved following the implementation of educational programs on opioid analgesia in 1990 in Taiwan [7].

However, pain in some patients did not improve but worsened, even under holistic care provided by a multidisciplinary team. Although the level of psychospiritual distress was not significantly different between the two groups upon admission, depression did not improve significantly one week after admission in the *not improved* group. Mori et al. recently reported on three advanced cancer patients with intractable pain, the cause of which was attributed to severe psychosocial distress [12]. Although the causality between depression and pain relief is hard to establish, our findings suggest that depression is an important psychological factor in determining whether cancer patients will experience effective pain management, especially when depression is difficult to manage. The reason why depression and pain are sometimes difficult to manage simultaneously may be associated with the individual’s psychosocial profile [12]. In addition to signs of physical deterioration, more attention should be directed to documenting over-time changes in psychospiritual distress. Successfully recognizing the risk factors underlying poor pain management, including both physical condition and psychospiritual distress, may be very important for effective cancer pain management strategies.

Pain is a complex multidimensional subjective experience and psychosocial components play an important role in cancer pain management [8]. Zaza and Baine systematically reviewed the relationship between cancer pain and psychological distress [23]. The authors found that increased pain was significantly associated with increased psychological distress [23]. Kane et al. and Kelsen et al. both reported that there was a significant cross-sectional association between pain and depression [24, 25]. Pain is a symptom in advanced cancer patients that is expressed in the same symptom pattern as depression [8]. Possible biological mechanism linking pain and depression is inflammation, such as elevated eosinophil counts [26]. Neuroimaging studies also reveal that brain activity, especially in the cingulate gyrus, is associated with pain, depression and social distress [27, 28], and the similar findings also exist in the cancer population [29, 30]. Recently, genetic researchers have reported that polymorphisms in some cytokines genes are potential markers for pain and depression in cancer patients [31, 32]. Psychospiritual factors linking pain and depression includes demoralization [33], loss of dignity [34], loss of hope [35], loss of help [36] and poor family/social support [37, 38]. These studies may support our findings.

Although pain and depression are highly prevalent in cancer patients [39] and literatures emphasize that pain and depression should be managed simultaneously for better outcomes [40], our study revealed more than 30 % of cancer patients still have unsatisfied pain control. Pain scores were low in the *not improved* group at admission, whereas this does not mean that pain was easy to treat with analgesics in these patients particularly when psychospiritual factors were difficult to manage. In the *not improved* group, depression and family/social support did not significantly improve. Demoralization, one of the troublesome psychological distress, is very common in cancer patients in Taiwan with the reported prevalence of 49.1 % [33]. Joblessness is associated with demoralization because it may cause a sense of uselessness [33]. Although most medical expenses of patients is paid by National Health Insurance which has been formed since 1995 [41], family caregivers still face the caring burden such as their own health problems, financial difficulties, and disruption of daily routine at home [42]; indeed, the caring burden of family certainly makes a significant impact on quality of life among terminally ill cancer patients [42]. These psychosocial factors make the management of cancer pain and depression more difficult.

The concept of total care provided by a palliative care team will result in an increased likelihood of improving depression, especially when pain is successfully controlled [43]. Furthermore, our results suggest that treating comorbid depression concomitantly with pharmacological and non-pharmacological managements may be beneficial in ameliorating pain. Most importantly, considering an individual’s psychosocial profile in cancer pain management is crucial, particularly when it is proving difficult to treat. The concept of *total pain*, pain consisting of physical, psychological, social and spiritual components, is very important in the care of advanced cancer patients [44]. Palliative and hospice care can continuously relieve psychosocial distress and fear of death while physical condition deteriorates gradually [21]. Unquestionably, patients with advanced cancer can have a better quality of life and experience a more peaceful death under palliative and hospice care [21].

Our study has some limitations. First, only individuals whose level of consciousness was clear enough (alert or lethargic consciousness) to report symptoms were recruited. Second, this study was conducted in a palliative care unit where active total care was provided. We did not assess other advanced cancer patients in other types of wards, or at home. Third, this is an observational study, and the findings therefore cannot confirm causality. Specifically, while many patients’ psychospiritual distress improved post admission, a number of patients saw no significant difference (*not improved* group). As such, while our results suggest that poor pain management may be associated with intractable depression, the inverse may equally be true, namely that addressing psychospiritual distress maybe impeded by intractable pain and symptom issues. Fourth, all participants in the study were Taiwanese, so the results should be confirmed in other ethic background. Fifth, our pain assessment tool was a single-dimensional numerical rating scale. A multidimensional tool such as the Melzack Pain Questionnaire will give more information related to the components of pain.

## Conclusion

There is a time-dependent relationship between pain relief and improvement of psychospiritual distress in advanced cancer patients. Routine assessment of psychospiritual distress factors should be considered in cancer pain management. More aggressive psychospiritual support may improve pharmacological pain management strategies in advanced cancer patients.

## Abbreviation

WHO: World Health Organization
